# *EFS *shows biallelic methylation in uveal melanoma with poor prognosis as well as tissue-specific methylation

**DOI:** 10.1186/1471-2407-11-380

**Published:** 2011-08-26

**Authors:** Lisa C Neumann, Andreas Weinhäusel, Stefanie Thomas, Bernhard Horsthemke, Dietmar R Lohmann, Michael Zeschnigk

**Affiliations:** 1 Institut für Humangenetik, Universitätsklinikum Essen, Hufelandstr. 55, 45157 Essen, Germany; 2 Austrian Research Center, Seibersdorf, Austria; 3 Augenklinik, Universitätsklinikum Essen, Essen, Germany

## Abstract

**Background:**

Uveal melanoma (UM) is a rare eye tumor. There are two classes of UM, which can be discriminated by the chromosome 3 status or global mRNA expression profile. Metastatic progression is predominantly originated from class II tumors or from tumors showing loss of an entire chromosome 3 (monosomy 3). We performed detailed *EFS *(*embryonal Fyn-associated substrate*) methylation analyses in UM, cultured uveal melanocytes and normal tissues, to explore the role of the differentially methylated *EFS *promoter region CpG island in tumor classification and metastatic progression.

**Methods:**

*EFS *methylation was determined by direct sequencing of PCR products from bisulfite-treated DNA or by sequence analysis of individual cloned PCR products. The results were associated with clinical features of tumors and tumor-related death of patients.

**Results:**

Analysis of 16 UM showed full methylation of the *EFS *CpG island in 8 (50%), no methylation in 5 (31%) and partial methylation in 3 (19%) tumors. Kaplan-Meier analysis revealed a higher risk of metastatic progression for tumors with *EFS *methylation (p = 0.02). This correlation was confirmed in an independent set of 24 randomly chosen tumors. Notably, only UM with *EFS *methylation gave rise to metastases. Real-time quantitative RT-PCR expression analysis revealed a significant inverse correlation of *EFS *mRNA expression with *EFS *methylation in UM. We further found that *EFS *methylation is tissue-specific with full methylation in peripheral blood cells, and no methylation in sperm, cultured primary fibroblasts and fetal muscle, kidney and brain. Adult brain samples, cultured melanocytes from the uveal tract, fetal liver and 3 of 4 buccal swab samples showed partial methylation. *EFS *methylation always affects both alleles in normal and tumor samples.

**Conclusions:**

Biallelic *EFS *methylation is likely to be the result of a site-directed methylation mechanism. Based on partial methylation as observed in cultured melanocytes we hypothesize that there might be methylated and unmethylated precursor cells located in the uveal tract. The *EFS *methylation of a UM may depend on which type of precursor cell the tumor originated from.

## Background

UM is the most frequent primary intraocular tumor in adults. Two classes of UM have been defined that differ in chromosome 3 status, metastatic risk and global mRNA expression profiles [1,2]. As tumors with monosomy 3 are tightly associated with metastatic progression, chromosome 3 testing is used to predict patients' prognosis [3,4]. Recently, inactivating somatic mutations in the gene encoding BRCA1-associated protein 1 (*BAP1*) on chromosome 3p21.1 were found to be frequent only in those UM that showed expression profiles linked to high metastatic potential [5]. One possible explanation for the clinical and genetic dichotomy of UM is distinct cell lineage, meaning that the two tumor classes stem from different melanocytic precursor cells located in the uveal tract [2,6]. In this regard, many examples are known where closely related terminally differentiated cells are characterized by distinct epigenetic patterns [7].

Most epigenetic studies in cancer focus on altered methylation of CpG islands (CGIs), which are found in the promoter regions of about 60% of all genes. With the exception of imprinted genes, genes on the inactive X-chromosome in females, germline-specific genes as well as a few developmental genes, the cytosine residues within CGIs > 500 bp are mostly unmethylated [8,9]. It is commonly assumed that epimutations, like other genetic changes in cancer, develop in a random manner and are then selected for growth advantage to the mutant cell clone. For example, hypermethylation of promoter-associated CGIs can result in transcriptional silencing of tumor suppressor genes (TSG) [10]. In these instances - in line with the model of two hit inactivation - one mutational hit alters the methylation pattern of one allele and the second allele is either lost or inactivated by a structural mutation. However, CGI methylation is not necessarily the result of an epimutation. In recent years, an increasing number of non-imprinted, autosomal CGIs and CpG-rich regions have been identified that are already methylated in non-neoplastic cells [8,9,11]. In some regions, this kind of CpG methylation establishes long-term gene inactivation and is part of the process of cell differentiation from pluripotent embryonic stem cells to terminally differentiated somatic cells [12-14]. This process finally results in a cell type specific methylation pattern [7]. A potential link between cell differentiation and cancer is suggested by the observation that genes that are preferentially hypermethylated in cancer are often marked for transcriptional repression through association with polycomb group proteins in embryonic stem cells [15,16].

Several methylation studies have been conducted to identify genes that, if hypermethylated, contribute to initiation and progression of UM [17-20]. Recently, we performed a comprehensive search for hypermethylation events in 16 UM using a screening assay based on methylation-sensitive restriction digest of genomic DNA followed by PCR amplification and array based detection of 323 different CGIs. Preliminary results of this screening revealed a CGI located in the promoter region of the *EFS *gene with a methylation pattern that is very unusual for TSGs. This prompted us to perform a detailed methylation analysis on the *EFS *CGI in UM and normal tissues.

## Methods

### Patients and specimens

Fresh tumor and peripheral blood samples were obtained from patients with UM treated at the Ophthalmology Department of the University Hospital of Essen by primary enucleation without prior radiation or chemotherapy. All patients were given diagnoses according to current ophthalmologic criteria. Follow-up data including tumor-related cause of death are available from all patients. All tumors analyzed in this study were selected from a cohort of 262 tumors with either monosomy 3 or disomy 3. The set of 16 tumors (set I) used to screen for and confirm altered methylation in UM was selected to equally represent tumors with monosomy 3 and disomy 3. The chromosome 3 status of all tumors was determined by microsatellite analysis [21]. The second set of 24 tumors (set II) was randomly chosen from the same cohort of 262 tumors, excluding tumor set I. Tumor and blood samples were stored at -80°C and -20°C, respectively. DNA from peripheral blood cells of patients and normal controls was extracted using the EZ1 DNA Blood 350 μl Kit (Qiagen). RNA and DNA purification from primary tumors was performed as described elsewhere [2]. Uveal melanocytes were isolated from eyes that were obtained from anonymous individuals not diagnosed with UM and were cultured in medium containing G418 at concentrations toxic for non-melanocytic cells [22]. Primary skin fibroblasts were obtained from healthy donors and cultivated as described elsewhere [23]. DNA from fibroblasts and buccal swabs of healthy donors was extracted using the EZ1 DNA Tissue Kit (Qiagen). DNA material from various human tissues was provided by Ralf Hermann (fetal brain), Dirk Prawitt (fetal liver, kidney and muscle), Bernhard Zabel (adult brain) and Osman El-Maarri (sperm). The research followed the tenets of the Declaration of Helsinki and was approved by the institutional ethical committee. Informed consent was obtained from the tumor patients after detailed explanation of the nature and possible consequences of the study.

### Methylation analysis

**Figure 1:**
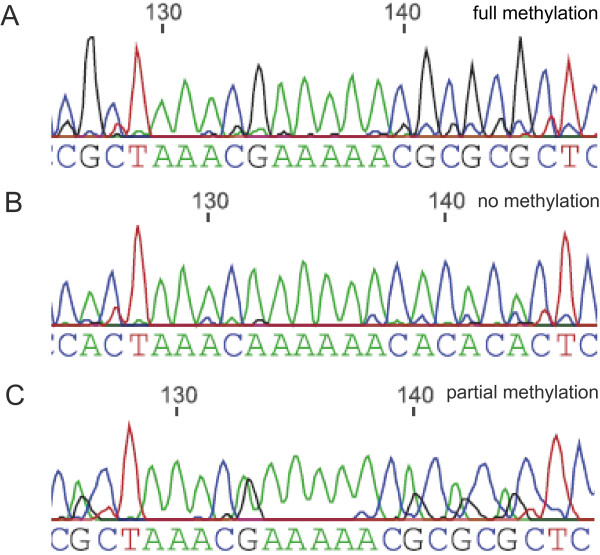
**Discrimination of methylation states based on direct bisulfite sequencing chromatograms**. Direct sequencing of PCR products from the *EFS *CpG island from bisulfite treated DNA. The reverse sequence is shown here. Therefore, the A signals represent unmethylated cytosines, which have been converted to thymine during bisulfite treatment of genomic DNA and the G signals represent methylated cytosines. (A) The analyzed region was classified as showing "full methylation" if A-signals were absent at every CpG; (B) "no methylation" in the absence of G signals at every CpG position or (C) "partial methylation" if both signals were present at some or all CpG positions.

Bisulfite modification of DNA was performed using an established protocol with minor modifications [24]. Genomic DNA (1-2 μg in 50 μl) was denatured for 15 min at 37°C by adding 5.5 μl of 3 M NaOH. For complete denaturation, samples were incubated at 95°C for 2 min and immediately cooled on ice. The bisulfite solution was freshly prepared by dissolving 4.25 g of sodium bisulfite (Sigma) in 7.5 ml H_2_O. 450 μl of 50 mM hydrochinone solution was added and the pH was adjusted to 5.15 by adding 0.5 ml of 10 M NaOH. The denatured DNA solution was mixed with 500 μl of the bisulfite solution and incubated at 50°C for 16-20 h in the dark. The DNA was recovered using the Wizard DNA Clean-Up System (Promega) followed by elution in 50 μl prewarmed H_2_O (65°C). Subsequently, 5.5 μl of 3 M NaOH was added and the samples were incubated for 15 min at 37°C. The solution was then neutralized by adding 55 μl of 6 M NH_4_OAc pH 7.0. The DNA was ethanol precipitated, washed in 70% ethanol, dried and resuspended in 15-30 μl of water depending on initial DNA input. To analyze the regions of interest, PCR was performed in a total volume of 25 μl containing 3 μl of bisulfite treated DNA, 0.2 mM of each dNTP, 0.2 μM of each primer, 2.5 μl 10x PCR-Puffer, 2.5 mM MgCl_2 _and 1.5 U Taq Polymerase (AmpliTaq Gold, Applied Biosystems, Foster City, CA) using a GeneAmp 9700 system. A touch down protocol was adopted as follows [25]: After denaturation at 95°C for 5 min, the annealing temperature was decreased 0.5°C every cycle from 63°C to 56°C, at which temperature 35 cycles were carried out. For all cycles, annealing was performed for 1 min, denaturation at 95°C for 20 sec and extension at 72°C for 1 min, followed by a final extension at 72°C for 5 min. To facilitate direct sequence analysis of PCR products from bisulfite-treated templates, GC-tagged primers were used containing additional nucleotides at the 5' end (indicated by bold letters) that do not bind to the template. The tags contain C and G nucleotides which are required for internal normalization during the Sanger sequencing [26]. Primer sequences: EFSfw: **CTTGCTTCCTGGCACGA**GTTTYGTTTTGGTTTTGTTTTAG; EFSrev: **TGTAAAACGACGGCCAGT**CATATTATCACTAAAACCAAAATCC. After agarose gel electrophoresis and purification using MinElute™ Gel Extraction Kit (Qiagen), sequence analysis was performed on an ABI 3100 automated capillary genetic analyzer (ABI) using Big Dye 1.1 (ABI) using a primer complementary to the tag sequence. *EFS *methylation was determined on the basis of all evaluable CpG positions in the analyzed region (11 CpGs located in the region chr14: 23835859-23835970 (GRCh37/hg19)). The analyzed region was classified as showing (i) full methylation if the T signal was absent or very low at every CpG position, (ii) no methylation in the absence of a C signal at every CpG position and (iii) partial methylation if both signals were present at some or all CpG positions (Figure 1). For sequence analysis of individual alleles, selected PCR products were cloned into the pGEM-T easy vector (Promega), according to manufacturer's instructions. This vector provides ready-to-use T-overhangs for ligation with A-overhangs of PCR-products. We cloned the same PCR products that had been analyzed by direct sequencing before and chose samples that showed partial methylation and that were informative for a SNP in the analyzed region (rs3759609). We picked 15-20 colonies per sample from which we isolated plasmid DNA using the Plasmid Mini Kit (Qiagen). The cloned fragments were sequenced using the SP6 Promoter Primer (Promega, Cat.# Q5011) and aligned with the help of the BDPC web interface http://biochem.jacobs-university.de/BDPC/) [27]. Sequences from cloned *EFS *PCR products were analyzed in their full lengths (28 CpG positions).

### Real-time quantitative PCR

*EFS *expression was analyzed by quantitative RT PCR using UPL-probe No. 17 (Universal probe library, Roche) and custom-designed primers (EFS-UPLProbe17-fw: TCCTGAACTGCCCGAGAG; EFS-UPLProbe17-rev: GCATTGCCCAGCATAGAAGT). We used specific probes and primers for Human *HPRT 1 *(*hypoxanthine phosphoribosyltransferase 1*) as reference gene (Human *HPRT1 *Hs99999909_m1, ABI). An aliquot of 0.5 μg total RNA was reverse transcribed using the GeneAmp RNA PCR Kit (ABI) according to the manufacturer's instructions. *EFS *quantitative RT-PCR was carried out in a volume of 20 μl containing 10 μl LightCycler 480 Probes Master, 2 μl of cDNA, 0.3 μM of UPL-probe No. 17 and 1 μM of each primer. Amplification was performed with the Applied Biosystems 7000 Real-Time PCR System under the following conditions: 95°C for 10 min followed by 40 cycles of denaturation at 95°C for 15 sec and annealing/elongation at 60°C for 1 min. The mean value of duplicate samples was used for further analysis. Relative expression levels were calculated by the comparative Ct Method as described in the tutorial "Guide to Performing Relative Quantitation of Gene Expression Using Real-Time Quantitative PCR" (Part #: 4371095 Rev B, Applied Biosystems).

Gene dosage analysis of *EFS *was performed by quantitative PCR using two independent assays located upstream (GRCh37/Hg19 position: chr14:23,836,138-23,836,198) and downstream (GRCh37/Hg19 position: chr14:23,835,737-23,835,837) of the region used for methylation analysis. UPL-probe No. 55 (Universal probe library, Roche) was used for measurement of the PCR product generated with custom-designed primers: EFS-AMP2-UPLProbe55-fw: GATGTGGGGCTAATGAAAGG and EFS- AMP2-UPLProbe55-rev: GGTCCGATCGGCTTTCTC. Primers EFS-AMP1-UPLProbe82-fw: AACTCCTGGTGGGGCTAGAT and EFS- AMP1-UPLProbe82-rev: GCTGGCACAAAAGTTGCTAGA were combined with UPL-probes No. 82. PCR was performed in a volume of 25 μl containing 12.5 μl PCR Master Mix without AmpErase (ABI), 15 ng DNA, 0.25 μM of UPL-probe and 0.4 μM of each primer. As a reference, we used a region on the proximal long arm of chromosome 15 (GRCh37/hg19 position: chr15:25166001-25166074) [28]. Amplification was performed with a LightCycler 480 System (Roche) under the same conditions as described for expression analysis. All samples were measured in duplicate, and the mean value was used for further analysis. Relative gene dosage levels were calculated by the delta-delta-C_P _method with the LightCycler 480 software (Roche) using standard curves and normal control blood DNA for all assays.

### Data analysis

Relationship between *EFS *expression and *EFS *methylation was evaluated using pair wise univariate chi-square test. Kaplan-Meier analysis was calculated using the Survival/Reliability tool of JMP^® ^statistical discovery software (JMP 7. SAS, Heidenheim, Germany). To determine the possible correlation of *EFS *methylation with clinical variables univariate analysis was performed using the chi-square test. The Log-Rank test was performed to test the probability that there is a trend in survival scores across two groups in Kaplan-Meier analysis.

## Results

We amplified the *EFS *CGI on bisulfite-treated DNA from 16 UMs (set I), cultured uveal tract melanocytes and 10 blood samples from healthy donors. Methylation of the CGI was determined by direct sequencing of the PCR products. This technique provides an average methylation measurement for each CpG across the DNA molecules of a given sample. The CGI was classified either as showing full methylation, no methylation or partial methylation (Figure 1). We found the *EFS *CGI fully methylated in 8 tumors, unmethylated in 5 tumors and partially methylated in 3 tumors. *EFS *was also fully methylated in blood DNA from healthy donors and partially methylated in DNA from cultured melanocytes isolated from the uveal tract. Interestingly, *EFS *was fully methylated in all but one tumor with monosomy 3. In contrast, full methylation was only found in one of eight tumors with disomy 3 (set I, Table 1).

**Table 1:** Clinical data of the patients included in the study

	Tumors									Patients					
	tumor ID	prominence [mm]	LBD [mm]	SBD [mm]	cell type	ciliary body involvement	chr.3 status	chr.8 alteration	*EFS *methylation	patient ID	age at diagnosis [years]	sex	survival	metastatic death	follow up time [months]
set I	T1	5.9	11.9	11.7	sc	no	D3	AI	no	28914	69	m	alive	-	51
	T2	5.8	11.2	10.6	sc	no	D3	AI	no	28101	69	f	alive	-	57
	T3	14.0	19.0	n.d.	sc	no	D3	AI	no	25722	48	f	alive	-	67
	T4	9.2	20.9	20.8	ec	no	D3	AI	partial	29311	39	m	deceased	yes	18
	T5	11.4	19.0	11.9	sc	no	D3	no	full	29802	66	f	alive	-	47
	T6	15.6	15.3	14.5	sc	no	D3	AI	no	22978	50	f	alive	-	81
	T7	9.2	10.0	8.7	sc	no	D3	AI	no	29101	64	f	alive	-	52
	T8	11.7	13.5	12.2	sc	no	D3	AI	partial	29380	72	m	alive	-	51
	T9	10.8	n.d.	n.d.	sc	no	M3	AI	full	30545	65	m	alive	-	46
	T10	12.0	13.4	11.9	sc	no	M3	no	partial	27745	67	f	deceased	no	45
	T11	10.3	21.9	21.1	mc	yes	M3	AI	full	24905	66	m	deceased	yes	26
	T12	9.8	17.0	13.5	mc	no	M3	AI	full	24903	64	f	deceased	yes	43
	T13	10.6	21.3	17.4	mc	no	M3	AI	full	25726	57	m	deceased	yes	19
	T14	9.8	25.0	21.0	sc	yes	M3	AI	full	24464	62	m	deceased	yes	22
	T15	11.3	15.5	11.6	sc	yes	M3	AI	full	27587	61	f	deceased	yes	20
	T16	8.5	n.d.	n.d.	sc	yes	M3	AI	full	25243	62	m	deceased	yes	18
set II	T17	7.1	11.2	10.6	sc	yes	D3	AI	no	14514	58	f	alive	-	125
	T18	9.9	16.0	11.9	sc	yes	D3	AI	no	17003	55	m	alive	-	112
	T19	10.4	15.5	14.4	sc	no	D3	AI	no	17453	19	f	alive	-	110
	T20	9.8	16.1	14.5	sc	yes	D3	AI	no	18671	46	f	alive	-	104
	T21	8.4	8.8	8.7	mc	no	D3	no	partial	19207	69	m	alive	-	102
	T22	n.d.	9.4	8.6	sc	no	D3	no	no	21436	38	m	alive	-	89
	T23	10.7	17.2	14.5	sc	no	D3	no	no	31091	63	m	alive	-	44
	T24	16.2	21.3	17.2	sc	no	D3	AI	no	31412	45	m	alive	-	39
	T25	6.0	13.4	12.1	sc	n.d.	D3	n.d.	no	33708	68	m	alive	-	29
	T26	12.5	12.1	9.8	sc	no	D3	no	no	36500	73	f	alive	-	20
	T27	12.0	22.7	18.9	sc	no	M3	AI	partial	17451	68	m	deceased	no	37
	T28	10.5	24.3	19.6	mc	yes	M3	no	full	18465	77	m	deceased	yes	37
	T29	12.3	16.0	15.2	mc	no	M3	AI	full	20012	76	m	deceased	yes	6
	T30	10.7	17.7	16.6	mc	yes	M3	AI	full	21501	71	m	deceased	yes	46
	T31	11.3	n.d.	n.d.	mc	yes	M3	AI	full	22095	74	m	deceased	yes	2
	T32	11.9	16.9	15.4	sc	no	M3	AI	full	22543	69	m	deceased	yes	5
	T33	9.3	20.0	n.d.	mc	no	M3	no	full	22805	70	m	deceased	no	18
	T34	10.6	15.8	15.5	sc	yes	M3	AI	full	25720	61	m	deceased	yes	50
	T35	13.7	n.d.	n.d.	sc	no	M3	AI	partial	26072	76	m	alive	-	61
	T36	11.3	11.4	7.0	mc	yes	M3	AI	full	27766	63	m	deceased	yes	35
	T37	10.1	12.9	10.7	sc	yes	M3	AI	full	29132	69	f	alive	-	52
	T38	10.1	20.4	16.5	sc	nd	M3	no	full	29337	74	f	alive	-	51
	T39	13.6	18.0	14.5	mc	no	M3	AI	full	32516	50	f	deceased	yes	30
	T40	8.3	12.5	11.5	mc	no	M3	AI	full	34922	54	f	alive	-	24

Methylation status of *EFS *as determined by direct sequencing of PCR products from bisulfite treated DNA; LBD, largest tumor diameter; SBD, smallest tumor diameter; M3, monosomy 3; D3, normal (retention of both alleles) chromosome 3 status; AI, allelic imbalance; m, male; f, female; sc, spindle cell; mc, mixed cell; ec, epitheloid cell type; n.d., not determined.

We analyzed the *EFS *genome dosage in all 8 tumors from set I that showed complete *EFS *methylation by quantitative RT-PCR to test whether complete methylation signals might be the result of monoallelic methylation on the background of a heterozygous *EFS *deletion. We found *EFS *genome dosage normal (two alleles) in all cases (data not shown), strongly suggesting biallelic *EFS *methylation.

**Figure 2:**
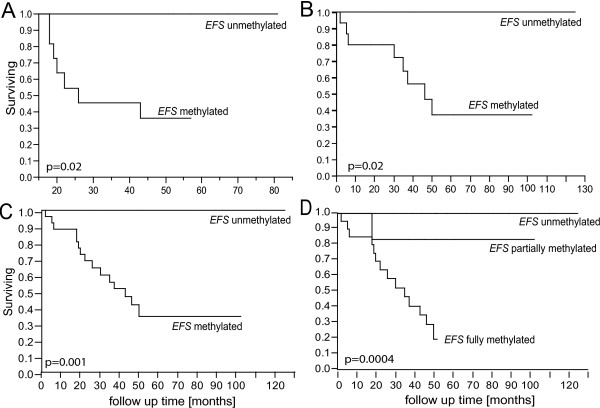
**Kaplan-Meier curves showing disease-specific mortality of UM patients**. Patients are grouped according to the *EFS *methylation status in their tumor. Partially and fully methylated samples are grouped together (A, B and C). (A) Set I: 16 patients used for the initial *EFS *CGI methylation analysis (p = 0.02). (B) Set II; confirmatory cohort of 24 randomly chosen tumor samples (p = 0.02). (C) Combined analysis of all 40 samples (setI + set II). (D) Combined analysis of all 40 samples (setI + set II) with partially methylated samples as separate group.

Kaplan-Meier analysis was used to compare the outcome of patients. Survival analysis revealed a reduced disease free survival of patients with complete or partial *EFS *methylation in their tumor (p = 0.02; Figure 2). However, as the number of patients in set I was rather small, we analyzed another set of 24 tumors randomly chosen from a cohort of 246 tumor samples showing either monosomy 3 or disomy 3. In this confirmatory group (set II), the significant correlation of *EFS *methylation and metastatic death of patients was confirmed (p = 0.02). Notably, only tumors with *EFS *methylation gave rise to metastases. The correlation is more significant when combined analysis of tumors from set I and set II (40 tumors) is performed (p = 0.001) and when tumors showing partial methylation are analyzed as separate group (p = 0.0004) (Figure 2).

Univariate analyses by pair wise comparison of *EFS *methylation with the variables listed in table 1 revealed a highly significant correlation of the *EFS *methylation with the chromosome 3 status (p = 1.6 × 10^-6^) and cell type (p = 0.006). For the continuous parameters of tumors such as LBD (largest basal diameter), SBD (smallest basal diameter), prominence and patients' age at diagnosis we used a logistic model and found that LBD and age at diagnosis reached significance with p = 0.03 and p = 0.02, respectively.

**Figure 3:**
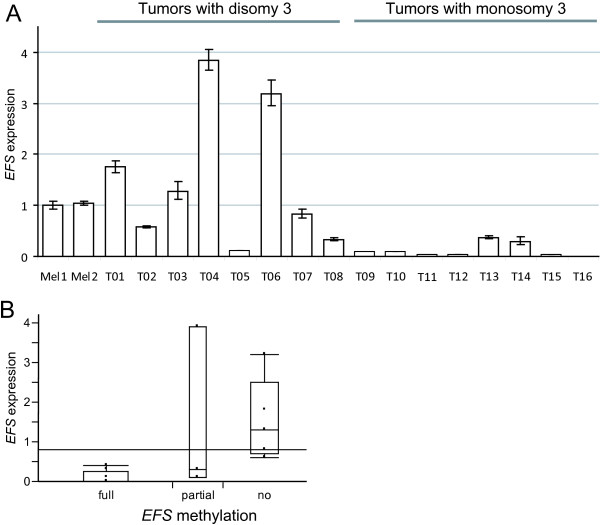
**Correlation of *EFS *expression and *EFS *methylation in 16 UM samples (set I)**. (A) *EFS *expression was normalized against *HPRT *(*Hypoxanthine-guanine phosphoribosyl-transferase*) expression and given relative to the *EFS *expression of cultured melanocytes (Mel1 and Mel2). (B) Box plot analysis of *EFS *expression in 16 UMs grouped according to the *EFS *methylation state. The difference in *EFS *expression between unmethylated and fully methylated tumor samples was statistically significant with p = 0.008 (Chi square). Differences between unmethylated and partially methylated tumors as well as between partially methylated and fully methylated tumors were not statistically significant.

We evaluated the possible correlation of *EFS *methylation and *EFS *expression by performing real-time quantitative RT-PCR on mRNA from the same set of tumors used for initial methylation analysis (set I; Figure 3). *EFS *expression was strongly reduced in tumors that showed complete *EFS *methylation compared to unmethylated tumor samples (p = 0.008).

To address the possibility of tissue-specific *EFS *methylation indicated by the distinct methylation in blood cells and melanocytes we performed methylation analysis of various normal tissue samples and primary cells (Table 2). *EFS *was unmethylated in all sperm samples, most fetal tissues, cultured primary fibroblasts and one of four buccal swab samples. Complete methylation was observed in 13 of 15 blood samples from UM patients. Two blood samples from UM patients (P_UM_1 and P_UM_15), three buccal swab samples from normal donors and all adult brain samples showed the presence of methylated and unmethylated signals at all CpG dinucleotides, and thus, were classified as partially methylated.

**Table 2:** *EFS *methylation in different tissues and cell types

Tissue/ cell type	Number of samples		Methylation	
		full	partial	no
Blood	10	10	0	0
Blood (patients)	15	13	2	0
Melanocytes	3	0	3	0
Fibroblasts	4	0	0	4
Sperm	2	0	0	2
Buccal swab	4	0	3	1
Brain (adult)	4	0	4	0
Brain (fetal)	1	0	0	1
Kidney (fetal)	1	0	0	1
Muscle (fetal)	1	0	0	1
Liver (fetal)	1	0	1	0
				

**Figure 4:**
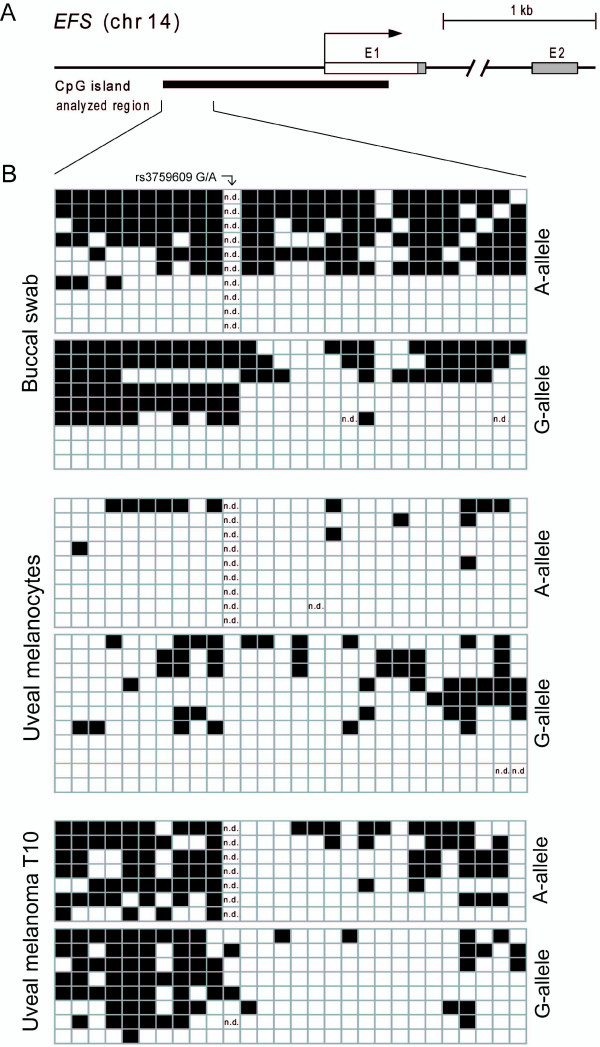
***EFS *CpG island analysis showing tissue-specific degrees of methylation**. (A) Map of the *EFS *promoter and exon1/exon2 region. CGI 131 (GRCh37/Hg19, black bar), which encompasses 144 CpG positions, overlaps with the *EFS *transcription start site (TSS). The analyzed region covers 260 bases of the CGI containing 27 CpG dinucleotides and is located upstream of the *EFS *TSS. The first analyzed CpG position is not part of the CGI (GRCh37/hg19). (B) Detailed methylation analysis of samples showing partial *EFS *methylation as determined by sequence analysis of cloned PCR products from one buccal swab sample, cultured uveal melanocytes and UM sample T10. Each row of boxes represents the CpGs of an individual PCR product. Each column represents a specific CpG in the analyzed region. Allelic discrimination was facilitated by an informative SNP (rs3759609). White boxes, unmethylated CpGs; black boxes, methylated CpGs; n.d., methylation state was not determined.

To evaluate the nature of partial methylation in more detail we cloned PCR products from representative samples with partial *EFS *methylation and sequenced individual clones (Figure 4). Cloned PCR products represent the methylation pattern of a single allele and might facilitate discrimination between allele-specific methylation and incomplete methylation of both alleles. We chose samples informative for a polymorphism in the analyzed region (SNP rs3759609), which allows for allele discrimination. In one buccal swab sample, both alleles were found either methylated or unmethylated suggesting the presence of a mixture of methylated and unmethylated cells. Incomplete, but biallelic methylation was found in UM sample T10. In cultured melanocytes, *EFS *methylation was less dense, but still affected both alleles. Allele-specific *EFS *methylation was found in none of the samples.

## Discussion

We found the methylation of the CGI located in the promoter region of *EFS *to be biallelic in all nine UM samples analyzed in this respect (eight fully methylated and one partially methylated UM from set I). Such frequent biallelic methylation is unlikely to occur through random epimutations and suggests the action of a site-directed *de novo *methylation mechanism, which is distinct from the two-step mutation-selection process that underlies allele-specific *de novo *hypermethylation of tumor suppressor genes. Tissue-specific methylation of *EFS *with full methylation in blood cells and partial or no methylation in most other tissues further argues for the involvement of such a methylation mechanism in the differentiation of the hematopoietic cell lineage. Site-specific methylation mechanisms, which may require the interaction of cis- and trans-acting factors, have been reported to be responsible for *de novo *methylation of tissue-specific methylated genes such as the *POU5F1 *pluripotency factor and are major components of cellular differentiation [12]. Most of the molecular components controlling this process are not yet known. With respect to UM, it would be interesting to find out if other genes are targeted by the same *de novo *methylation mechanism responsible for *EFS *methylation. Identification of these genes might provide further insight into the biological difference inherent to both uveal melanoma classes as methylation is strongly associated with metastatic progression.

The strong correlation of *EFS *methylation with transcriptional *EFS *silencing observed in UM shows that *EFS *is subjected to long term inactivation by the methylation mechanism. The *EFS *promoter might therefore belong to the group of tissue-specific differentially methylated regions that contribute to tissue-specific transcription control [29].

We detected partial methylation in DNA from some normal tissue samples. Tissue heterogeneity might contribute to this observation as blood cells, which are methylated at *EFS*, are likely to contribute to the DNA extracted from some tissue samples. Partial methylation was also found in cultured uveal melanocytes. As these cells were grown in geneticin at a concentration toxic for non-melanocytic cells, methylated *EFS *alleles are likely to be derived from melanocytes [22]. We cannot exclude that *EFS *methylation in these cells may have arisen in the course of cultivation. However, no *EFS *methylation was detected in cultured primary fibroblasts indicating that growing primary cells under cell culture conditions *per se *is not sufficient to induce *EFS *methylation. Analysis of individual clones representing individual alleles revealed CGI methylation of both parental *EFS *alleles (A and G allele; Figure 4B.), which argues for the existence of both methylated and unmethylated melanocytes in the uveal tract.

Genome and transcriptome analyses have shown that UMs fall into two classes that are congruent with high and low risk of death from metastatic disease [1,2]. Here we found that *EFS *methylation is significantly correlated with patients' survival and the chromosome 3 status of the tumors. In fact, all patients in our series who died of metastases showed *EFS *methylation in their primary tumors. Correlation of *EFS *methylation, although to a lesser extent, was also observed with the cell type, age at diagnosis and LBD. These parameters have previously been shown to correlate with monosomy 3 in UM suggesting that *EFS *methylation is a typical feature of the tumor class characterized by monosomy 3.

From our data we cannot decide if the *EFS *methylation state was already present in tumor precursors or if it was acquired during tumorigenesis. Taking the differentially methylated melanocyte precursors into account, we propose that tumors with *EFS *methylation might be direct descendants from methylated precursors. Further progression of these tumors into a highly malignant tumor phenotype might frequently, but not necessarily, be accompanied by loss of a chromosome 3. This is in agreement with the idea of different melanocytic precursors as origin of the different classes of UM previously published by Tschentscher *et al*. and Chang *et al*. [2,6]

Although EFS has been recognized as a member of the CAS protein family, little is known about its function so far. The protein contains a Src homology 3 (SH3) domain and has first been identified by interactions with the Src-family kinases Src, Fyn and Yes in mice [30,31]. Like other CAS family members EFS seems to act as an adapter protein, which is regulated by phosphorylation but has no enzymatic activities [32]. It was shown to be expressed in the thymic stroma [33] where it is involved in T-lymphocyte regulation and *EFS*-knockout mice develop exaggerated T-cell responses and inflammatory lesions [34]. EFS has not been linked to cancer, so far. However, other members of the CAS family can act as oncogenes and have been linked to metastasis and poor prognosis [32]. Involvement of the CAS proteins BCAR1 and NEDD9 in focal adhesions and mitotic spindle assembly, respectively, might hint towards implications of *EFS *in UM invasion or chromosome 3 loss.

## Conclusions

Biallelic *EFS *methylation hints towards a site-specific methylation mechanism. Partial, but biallelic methylation in cultured melanocytes from the uveal tract suggests that there are methylated and unmethylated precursor cells. Therefore, *EFS *methylation of an UM may depend on which type of precursor cell the tumor originated from. It remains to be elucidated whether biallelic *EFS *methylation is established during progression of UM, or whether it mainly represents an early epigenetic flag that traces the two tumor classes back to their precursor cell. Identifying other genes targeted by the site-specific methylation mechanism might provide further insight into the biological difference underlying both UM classes.

## Competing interests

The authors declare that they have no competing interests.

## Authors' contributions

LCN carried out most of the experiments and wrote the manuscript. AW carried out the preliminary hypermethylation study. ST supervised patients care and was involved in collecting follow up data. BH and DRL participated in the design of the study and helped to write the manuscript. MZ designed the study, performed the statistical analysis and wrote the manuscript. All authors read and approved the final manuscript.

## Pre-publication history

The pre-publication history for this paper can be accessed here:

http://www.biomedcentral.com/1471-2407/11/380/prepub
